# Therapeutic Review for 1880

**Published:** 1881-03

**Authors:** 


### THERAPEUTIC REVIEW FOR 1880.

As usual, a very considerable number of new drugs and preparations, as well as the revival of many old favorites, require to be mentioned. Thus, pulsatilla has been recommended for dysmenorrhea, and for the headache of exhaustion; the rinacanthus communis for ringworm; and muscarin (*amanita muscaria*) for night-sweats in phthisis. Resorcin appears to possess decidedly antiseptic properties; but both the amount of excitement which it produces and the briefness of its effect are drawbacks to its general adoption in treatment of fever. Fuchsin, in doses of one to three grains in twenty-four hours, has been found to reduce the amount of albumen in some cases of Bright's disease. The oxalate of cerium in the cough of phthisis, bronchitis, pertussis and other conditions has proved useful chiefly as an indirect, but probably also as a direct, pulmonary or respiratory sedative. Benzoate of soda, which last year received its *coup de grace* as a remedy for phthisis, has lately been praised in the treatment of whooping-cough, scarlet fever, diphtheria and gonorrheal ophthalmia. On the other hand, it is claimed that good results have followed the administration of crude petroleum (in capsules) in chronic diseases of the lungs. Chloride of calcium appears to have been used with benefit in some forms of phthisis. Caffein, in the form of citrate, has been proved to deserve a trial as a diuretic in cardiac dropsy, if other measures fail. Whatever may be the value of pyrogallic acid in the treatment of scaly diseases of the skin, it certainly must be used with caution, as the application of it to an extensive surface has caused death in at least one instance by dissolution of the blood corpuscles. Quebracho bark maintains its good character as a remedy for dyspnea from pulmonary or cardiac disease, and in spasmodic asthma. The action of the nitrites of sodium and potassium has been quite recently investigated in America and Germany, and the results appear to show that in these drugs we possess remedial agents which at once possess many of the most important properties of nitrite of amyl, and are more gradual and persistent in their influence.

As an antiseptic, boracic acid has been recommended by certain Continental and American authorities; benzoic acid by others; a solution of acetate of alumina has been highly recommended in Germany, both for its activity and for its cheapness; and the salts of copper are said to possess the same properties by Dr. Burq. Another claimant for trial and attention as an antiseptic has been brought forward in Edinburgh in menthol, a crystalline solid derived from the essential oil of peppermint; and this substance is said to be also "anti-neuralgic."

Of Chian turpentine, it must be said with regret, though certainly without surprise, that it has proved in the hands of the most trustworthy observers to be utterly valueless as a "cure" for cancer, and to have seldom any influence over a single symptom of the disease.

Nitro-glycerine has been found to be a drug of real value in many, although naturally not in all, cases of angina pectoris; and there is considerable evidence in favor of its use in sea-sickness. Jaborandi and its active principle, pilocarpin, have been tried without more than very moderate success in hydrophobia; with small success in perspiratory disorders of the skin, psoriasis and acute eczema; and with more encouraging results in prurigo, chronic urticaria, chronic eczema, various forms of asthma, in myxedema and in Bright's disease. It appears, also, to act occasionally as a useful galactogogue. From America we hear that the hypodermic injection of pilocarpin has proved to be of great value in the paroxysm of ague, when given immediately before the attack, or in the cold stage; and it is alleged that when the incipient symptoms are thus checked, the malarial disease may be actually relieved for a time, whether with or without the administration of quinine in the intervals. The stigma of the maize has been highly spoken of in France as a diuretic and sedative to the genito-urinary tract, especially in calculus.

Gelseminum has come into more general use during the year in the treatment of the acute periods of neuroses, especially of neuralgia and megrim. Bromide of ethyl has been employed both as a general and as a local anesthetic, but unfortunately not always with success, or even safety. Iodide of ethyl has been highly praised as an anti-spasmodic in asthma.

During the year a considerable number of cases have been reported in which ergot has appeared to be of benefit in diabetes and diabetes insipidus. A new mydriatic, called homatropin, has been introduced, which promises to be equally certain in its action on the eye with atropin, and at the same time less irritant, whilst its effect pass off more quickly. Professor Ladenburg, of Riel, has proved that the alkaloids called hyoscyamin, daturin and duboisin are identical with each other, and isomeric with atropin, into which they may be converted.

Acupuncture, which seems lately to have fallen into comparative disrepute, has been re-introduced to the profession as a really valuable remedy for neuralgia by Mr. Snell. At the same time many important instances have been recorded during the year of the unquestionable effects of the application of pieces of metal, wood and other substances to the surface of the body in hysteria and hystero-epilepsy; and the intelligent therapeutist will do well 'to hesitate before he discards this method of treatment as imaginary and useless.

Various preparations of petroleum, etc., which do not require to be particularized, have been introduced as rivals of vaseline; and the use of these bodies as a basis for ointments instead of animal fat is rapidly extending.—*Medical Times and Gazette.*

